# Supplementary material for: Hi-C metagenome sequencing reveals soil phage–host interactions
Source: Nat Commun. 2023 Nov 23;14:7666. doi: 10.1038/s41467-023-42967-z (PMC10667309; doi:10.1038/s41467-023-42967-z)
Supplement: Supplementary file 1 — Supplementary Information [file 41467_2023_42967_MOESM1_ESM.pdf]

Supplementary information

Hi-C Metagenome Sequencing Reveals Soil Phage-Host Interactions

Ruonan Wu[1], Michelle R. Davison[1], William C. Nelson[1], Montana L. Smith[1], Mary S. Lipton[1], Janet K. Jansson[1], Ryan S. McClure[1], Jason E. McDermott[1,2], Kirsten S. Hofmockel[1,3]*

[1]Earth and Biological Sciences Directorate, Pacific Northwest National Laboratory, Richland, WA, USA

[2]Department of Molecular Microbiology and Immunology, Oregon Health & Science University, Portland, OR, USA

[3]Department of Agronomy, Iowa State University, Ames, IA, USA

*Corresponding author: kirsten.hofmockel@pnnl.gov

Keywords: soil bacteriophage, phage-host interactions, High-Throughput Chromosomal Conformation Capture (Hi-C), metagenomics, metatranscriptomics

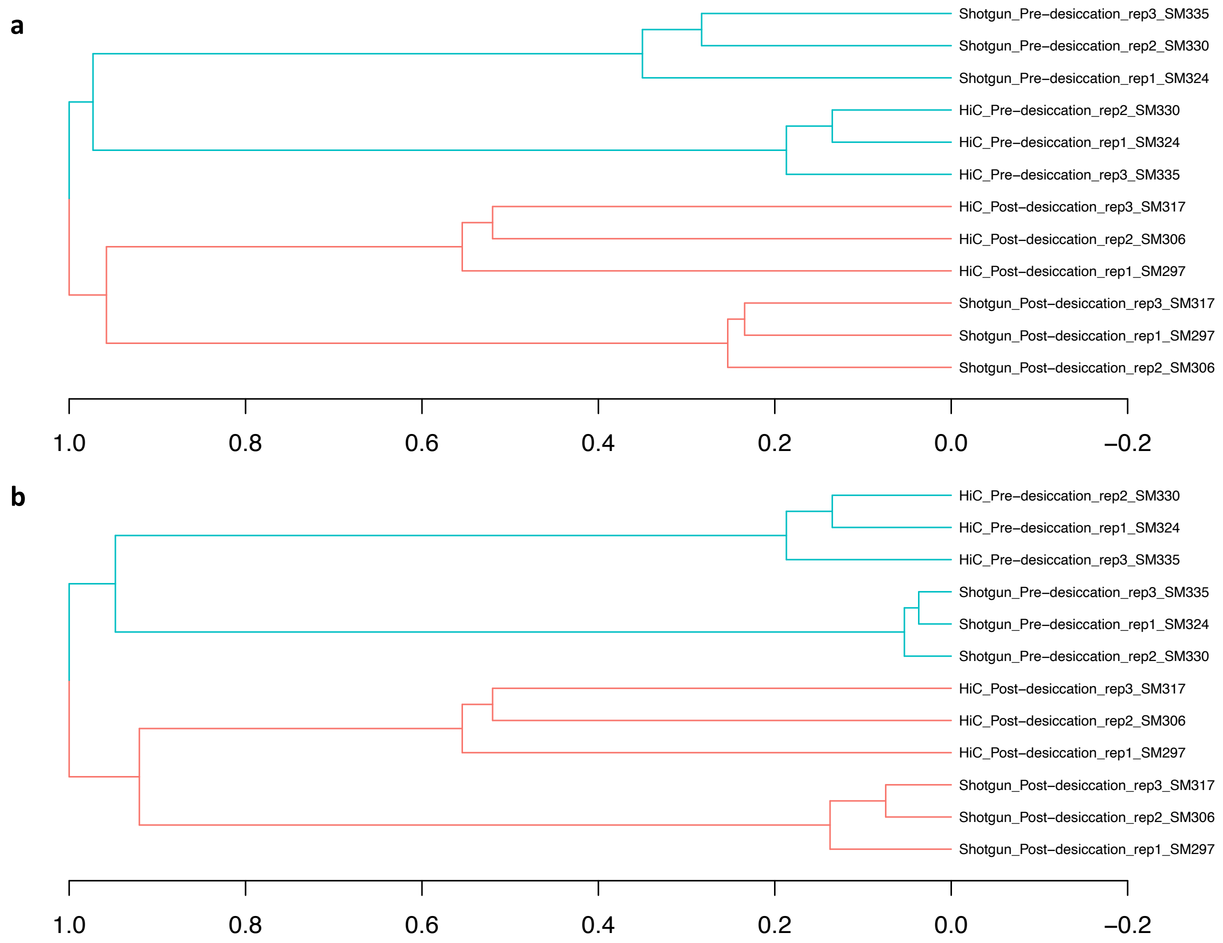

Figure S1. Hierarchical clustering of the viral communities recovered by Hi-C and shotgun metagenomes before and after soil drying. The viral communities detected in shotgun metagenomes (labeled as 'shotgun') including all the identified vOTUs (a) or including only the vOTUs that contain host-associated viruses (b) were clustered with the viral communities detected in the Hi-C metagenomes (labeled as 'HiC'). The Bray-Curtis distance matrix of the detected communities was evaluated by a multivariate analysis of variance with a permutation test (999 permutations). The recovered viral communities were significantly impacted by soil drying ($p \leq 0.005$). The Hierarchical clustering was based on the pairwise Bray-Curtis distances among the detected viral communities. The edges representing viral communities recovered before and after soil drying are colored blue and red, respectively.

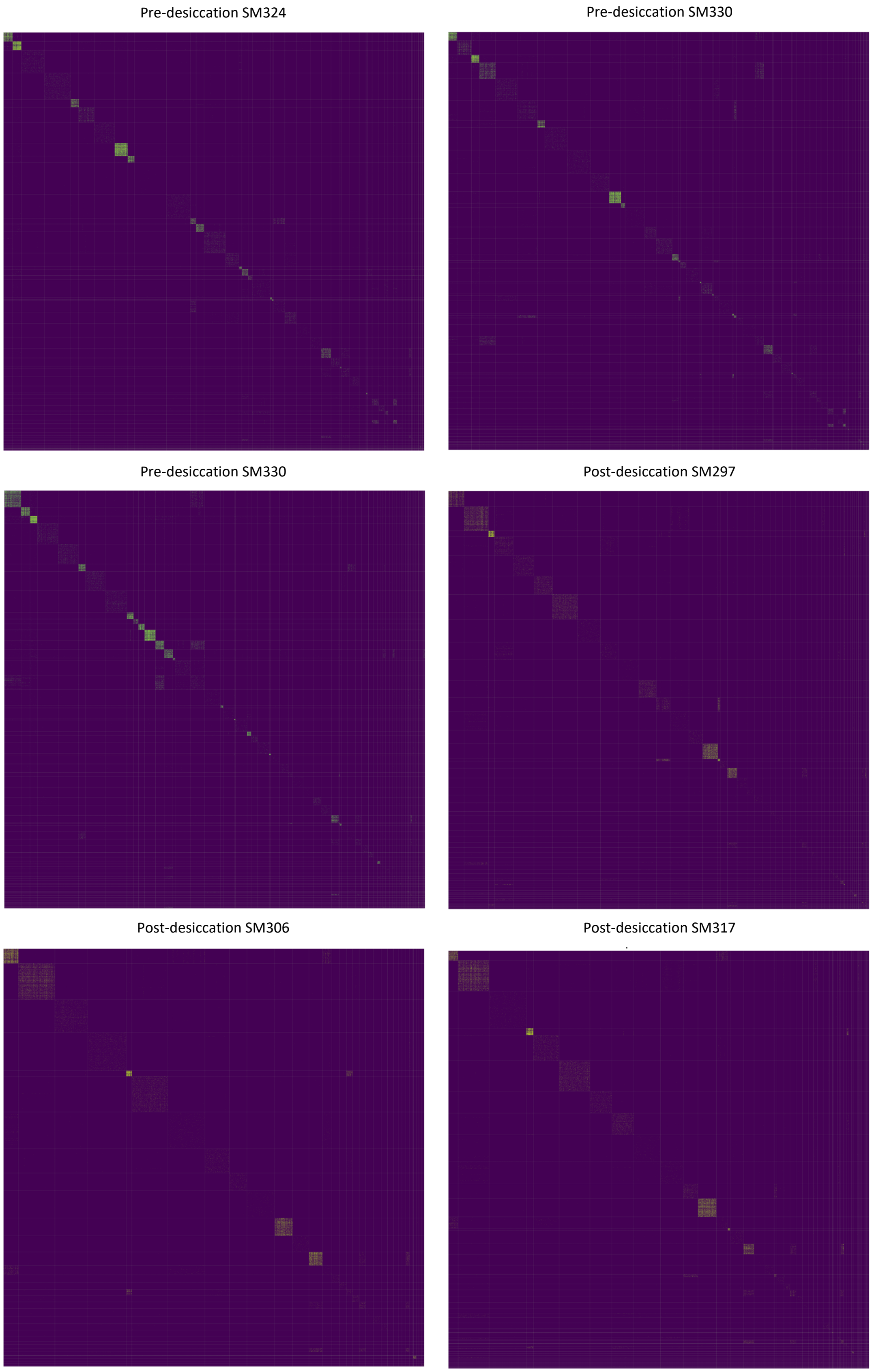
Pre-desiccation SM324
Pre-desiccation SM330
Pre-desiccation SM330
Post-desiccation SM297
Post-desiccation SM306
Post-desiccation SM317

Figure S2. Hi-C contact maps. Hi-C metagenomic contact map was generated for each of the three replicate samples before (SM324, SM330, SM335) or after soil desiccation (SM297, SM306, and SM317). The contact map axes symmetrically depict the binned contigs grouped by bin assignment, delineated with dashed gray lines, and ordered by bin extent. Cell Intensity indicates the log-scaled Hi-C contact counts between pairs of binned contigs, using a smoothing value of 1E-10. Diagonal values, where the contigs in the pair are equal, are set to zero to better visualize the inter-contig signal.

Table S1. Top 10 most central nodes in a host co-abundance network and top 10 most abundant MAGs. MAGs highlighted with asterisks (*) are abundant and central.

Highly Central Nodes	Highly Abundant Contigs
drep_102*	drep_7*
drep_117	drep_102*
drep_38	drep_79*
drep_43	drep_106
drep_63	drep_5
drep_7*	drep_75*
drep_75*	drep_15
drep_79*	drep_2
drep_8	drep_3
drep_89	drep_131
