# Supplementary material for: Hi-C metagenome sequencing reveals soil phage–host interactions
Source: Nat Commun. 2023 Nov 23;14:7666. doi: 10.1038/s41467-023-42967-z (PMC10667309; doi:10.1038/s41467-023-42967-z)
Supplement: Supplementary file 3 — Description of Additional Supplementary Files [file 41467_2023_42967_MOESM3_ESM.pdf]

Description of Additional Supplementary Files

Supplementary Data 1: Sequencing statistics and quality matrices of the five released datasets. Supplementary Data 1A Sequencing depth of the shotgun metagenomes; Supplementary Data 1B Assembly statistics of shotgun metagenomes; Supplementary Data 1C Sequencing depth of Hi-C metagenomes and the quality matrix of Hi-C reads mapping back to the shotgun metagenome assemblies; Supplementary Data 1D Description of the data package including the identified phage-host links; Supplementary Data 1E Sequencing depth of the metatranscriptomes. The five datasets are packaged and minted with DOIs.

Supplementary Data 2: Dereplicated viral contigs detected from metagenome assemblies. The dataset includes length, vOTU assignment and results from different bioinformatic tools (Demovir[67], DeepVirFinder[53], VirSorter[51], VIBRANT[52] and CheckV[54]) for each of the dereplicated viral contigs identified from metagenome assemblies.

Supplementary Data 3: Coverage estimates for viral contigs in replicate metagenomes. Breadth and depth of coverage of each of the dereplicated viral genomes in the replicate metagenomes were calculated. When a viral contig had a breadth of coverage in a metagenome lower than 50% (highlighted with asterisks or '*'), the respective depth of the coverage was adjusted to zero.

Supplementary Data 4: Read coverage and density of all unfiltered linkages detected in by Hi-C metagenome sequencing. The statistics of all unfiltered linkages include the raw and processed contig read depth, the linked contig read depth, intra- and inter-linkage read count and density (with and without normalization) in Hi-C metagenomes generated from each replicate sample.

Supplementary Data 5: Phage-host pairs informed by Hi-C metagenome sequencing. The detected phage-host pairs were quality filtered according to the method described in Uritskiy et al. 2021⁷³ (details in Methods).

Supplementary Data 6: Taxonomic assignments of the metagenome-assembled genomes (MAGs). Data include the binning statistics, clustering results and taxonomic assignments of the MAGs.

Supplementary Data 7: Phage-host links predicted using the CRISPR spacer matching method. The phage contigs were matched to the CRISPR spacers retrieved from the MAGs.

Supplementary Data 8: Node statistics for the microbial co-occurrence networks. The centrality indices of each node (representing a dereplicated MAG) within the three microbial co-occurrence networks are shown.

Supplementary Data 9: Differential abundances in *argS* gene transcripts. A total of 30 metatranscriptomes were generated along the course of soil pre-incubation and desiccation. The transcript abundances of *argS* genes were calculated from each of the metatranscriptome data and normalized across the datasets.
