# Supplementary material for: Hi-C metagenome sequencing reveals soil phage–host interactions
Source: Nat Commun. 2023 Nov 23;14:7666. doi: 10.1038/s41467-023-42967-z (PMC10667309; doi:10.1038/s41467-023-42967-z)
Supplement: Supplementary file 13 — Reporting Summary [file 41467_2023_42967_MOESM13_ESM.pdf]

nature portfolio

Corresponding author(s): Kirsten Hofmockel
Last updated by author(s): Oct 25, 2023

Reporting Summary

Nature Portfolio wishes to improve the reproducibility of the work that we publish. This form provides structure for consistency and transparency in reporting. For further information on Nature Portfolio policies, see our Editorial Policies and the Editorial Policy Checklist.

Statistics

For all statistical analyses, confirm that the following items are present in the figure legend, table legend, main text, or Methods section.

n/a	Confirmed	
☐	☒	The exact sample size (*n*) for each experimental group/condition, given as a discrete number and unit of measurement
☐	☒	A statement on whether measurements were taken from distinct samples or whether the same sample was measured repeatedly
☐	☒	The statistical test(s) used AND whether they are one- or two-sided<br>*Only common tests should be described solely by name; describe more complex techniques in the Methods section.*
☒	☐	A description of all covariates tested
☒	☐	A description of any assumptions or corrections, such as tests of normality and adjustment for multiple comparisons
☐	☒	A full description of the statistical parameters including central tendency (e.g. means) or other basic estimates (e.g. regression coefficient) AND variation (e.g. standard deviation) or associated estimates of uncertainty (e.g. confidence intervals)
☐	☒	For null hypothesis testing, the test statistic (e.g. *F*, *t*, *r*) with confidence intervals, effect sizes, degrees of freedom and *P* value noted<br>*Give P values as exact values whenever suitable.*
☒	☐	For Bayesian analysis, information on the choice of priors and Markov chain Monte Carlo settings
☒	☐	For hierarchical and complex designs, identification of the appropriate level for tests and full reporting of outcomes
☐	☒	Estimates of effect sizes (e.g. Cohen's *d*, Pearson's *r*), indicating how they were calculated

Our web collection on statistics for biologists contains articles on many of the points above.

Software and code

Policy information about availability of computer code

Data collection	The sequencing data were exported from the sequencer, Illumina NovaSeq. No other software was used to collect the data.
Data analysis	the listed softwares/tools/algorithms/packages were used to analyze to data which are all public available: fastp (v0.20.1), MEGAHIT (v1.2.9),BWA-MEM (v0.7.17),SAMBLASTER (v0.1.24), samtools (v1.9), CheckM (v1.2.0), dRep (v3.4.0), GTDB-Tk (v2.1.0),VirSorter (v2), VIBRANT (v1.2.1), DeepVirfinder (v 2020.11.21), CheckV (v0.7.0) , ViPTreeGen (v1.1.2), vConTACT2 (v0.9.19), BBMap (v 38.34),BamM (v1.7.3),samtools (v1.9), R(v4.2.0), CRISPRCasFinder (v3.1.0), RStudio(v2022.07.1), bin3C (v0.11), Markov chain Monte Carlo (MCMC)-based algorithm, Demovir workflow (https://github.com/feargalr/Demovir).<br>R code is deposited at Github, https://github.com/Ruonan0101/SFA_Hi_C_MS.

For manuscripts utilizing custom algorithms or software that are central to the research but not yet described in published literature, software must be made available to editors and reviewers. We strongly encourage code deposition in a community repository (e.g. GitHub). See the Nature Portfolio guidelines for submitting code & software for further information.

Data

Policy information about availability of data

All manuscripts must include a data availability statement. This statement should provide the following information, where applicable:

- Accession codes, unique identifiers, or web links for publicly available datasets
- A description of any restrictions on data availability
- For clinical datasets or third party data, please ensure that the statement adheres to our policy

The raw or large processed data generated in this study have been packaged and publicly available at DataHub (https://data.pnnl.gov/group/nodes/dataset/33511) as well as NCBI under BioProject PRJNA1006511. Four data packages include: 1) raw sequences of the six shotgun metagenomes (DOI: 10.25584/1922087, https://data.pnnl.gov/group/nodes/dataset/33337, NCBI: SRR25682926- SRR25682930), 2) all the shotgun metagenome-assembled contigs (>1 kb), MAGs and the dereplicated viral contigs (DOI: 10.25584/1922088, https://data.pnnl.gov/group/nodes/dataset/33338, NCBI: JAWMQX000000000 and JAWMQY000000000 for the contigs assembled from soils with pre- or post-desiccation treatment), 3) the unique phage-host pairs detected by Hi-C (DOI: 10.25584/1922090, https://data.pnnl.gov/group/nodes/dataset/33339), 4) the quality-filtered metatranscriptomes (DOI: 10.25584/1922091, https://data.pnnl.gov/group/nodes/dataset/33340, NCBI: SRR25916027- SRR25916064) and 5) raw sequences of the six Hi-C metagenomes (DOI: 10.25584/1970740, https://data.pnnl.gov/group/nodes/dataset/33511, NCBI: SRR25689209-SRR25689214). A detailed description of each data package is in Supplementary Data 1. Source data are provided with this paper.

Human research participants

Policy information about studies involving human research participants and Sex and Gender in Research.

Reporting on sex and gender	not applied
Population characteristics	not applied
Recruitment	not applied
Ethics oversight	not applied

Note that full information on the approval of the study protocol must also be provided in the manuscript.

Field-specific reporting

Please select the one below that is the best fit for your research. If you are not sure, read the appropriate sections before making your selection.

☐ Life sciences ☐ Behavioural & social sciences ☒ Ecological, evolutionary & environmental sciences

For a reference copy of the document with all sections, see nature.com/documents/nr-reporting-summary-flat.pdf

Ecological, evolutionary & environmental sciences study design

All studies must disclose on these points even when the disclosure is negative.

Study description	This study demonstrates that shifts in soil moisture have dramatic impacts on phage-host interactions and thus on the host population dynamics and community structure stability by applying Hi-C approach to chemically link phages and the infected hosts and integrating multi-omics (metagenome and metatranscriptome). The study includes pre and post desiccation samples, each with 3 biological replicates.
Research sample	Surface soils (0-20 cm deep, 5 cm diameter cores) were collected from 16 randomly selected locations immediately adjacent to the Tall Wheatgrass Irrigation Field Trial experiment in Prosser, WA (46°15'04"N, 119°43'43"W) in June 2020. The field samples were chosen because they represent typical marginal grassland soils that are commonly exposed to changes in moisture and climate impact.
Sampling strategy	Soil microcosms were prepared for each treatment in three replicates. The three wet soil samples were collected when the soil reached to 75% water holding capacity after pre-incubation. The three dry soil samples were collected after the soils were desiccated after 14-day's soil drying. Three replicate samples will enable us to test statistically to detect treatment differences. No specific sample size calculations were performed.
Data collection	DNA and RNA used for shotgun metagenomes and metatranscriptomes were extracted from 0.25 g and 2 g, respectively, of soils in each microcosm using PowerSoil DNA and RNA extraction kits according to the manufacturers' instructions. This was performed by PNNL staff. Shotgun metagenome sequencing was performed by Phase genomics on an Illumina NovaSeq generating an average of 177 million PE150 read pairs. Metatranscriptome sequencing was performed on an Illumina NovaSeq S4 by JGI.<br>The cross-linked DNA extracted from 5 g of soil in each replicate microcosm was digested using Sau3AI and MlucI restriction enzymes, and proximity-ligated with biotinylated nucleotides to create chimeric molecules. The chimeric molecules were pulled down with streptavidin beads and collected for ProxiMeta library preparation by Phase Genomics. The Hi-C metagenome sequencing

	was performed on an Illumina NovaSeq by Phase Genomics.
Timing and spatial scale	All samples were collected and processed in June 2020. This was a one time field collection. Incubation were conducted and harvested over a 2 week incubation in June 2020. All subsequent data were generated at one instance with no periodicity. Samples were transported to the Pacific Northwest National Laboratory (PNNL) on ice and stored at 4℃until processing. After homogenizing the soils and measuring soil moistures, sixty grams (dry weight soil equivalent) were weighed into autoclaved 4 oz jars and the soils were brought to 75% water holding capacity by addition of sterile deionized water. Three replicate incubations were set up for each of 2 sampling time points (n=6). First, the soils were preincubated for one week during which time they were gradually exposed to increasing temperature until the target incubation temperature of 30℃ average summer temperature (https://www.usclimatedata.com/climate/prosser/washington/united-states/uswa0355) was reached and harvested representing the wet soils. The remaining 3 incubation jars continued to incubate at 30℃, without any further moisture additions. Moisture loss was monitored by mass, until complete desiccation was achieved after 14 days. Then the final 3 replicate samples were harvested representing the dry soils. The six soil samples with three from each timepoint were stored at -80℃ until processed for DNA and RNA extractions.
Data exclusions	No data were excluded.
Reproducibility	Three biological replicates were included for each moisture treatment and sequenced separately. All attempts at replication were successful.
Randomization	Surface soils (0-20 cm deep, 5 cm diameter cores) were collected from 16 randomly selected locations immediately adjacent to the Tall Wheatgrass Irrigation Field Trial experiment in Prosser, WA (46°15'04"N, 119°43'43"W).<br>Randomization is not applicable to the microcosm experiment in this study because we are dealing with a small number of samples.
Blinding	Blinding is not applicable to this study because it was necessary to track the individual sample ids during the experiments.

Did the study involve field work? ☒ Yes ☐ No

Field work, collection and transport

Field conditions	Samples were collected from 16 randomly selected locations immediately adjacent to the Tall Wheatgrass Irrigation Field Trial experiment in Prosser, WA (46°15'04"N, 119°43'43"W) in June 2020. This field site is an arid grassland planted with Tall Wheatgrass (Thinopyrum ponticum). Soils are aridisols reflective of the hot and dry climate. Average summer temperature is 30 degrees C and average annual precipitation is 180 mm.
Location	Samles were collected from 16 randomly selected locations immediately adjacent to the Tall Wheatgrass Irrigation Field Trial experiment in Prosser, WA (46°15'04"N, 119°43'43"W) in June 2020.
Access & import/export	All samples were collected in compliance with local, national and international laws. Soils are unregulated. Soils were collected in collaboration and compliance with the Washington State University Irrigated Agriculture Research and Extension Center. All labs handing soils were permitted appropriately.
Disturbance	Appropriate access roads were used to collect samples. Minimal disturbance was incurred to maintain the integrity of this long term field experiment.

Reporting for specific materials, systems and methods

We require information from authors about some types of materials, experimental systems and methods used in many studies. Here, indicate whether each material, system or method listed is relevant to your study. If you are not sure if a list item applies to your research, read the appropriate section before selecting a response.

Materials & experimental systems

n/a	Involved in the study
☒	☐ Antibodies
☒	☐ Eukaryotic cell lines
☒	☐ Palaeontology and archaeology
☒	☐ Animals and other organisms
☒	☐ Clinical data
☒	☐ Dual use research of concern

Methods

n/a	Involved in the study
☒	☐ ChIP-seq
☒	☐ Flow cytometry
☒	☐ MRI-based neuroimaging
